# Engineered Zn_0.2_Fe_2.8_O_4_@Cu(II)-Based Core@Shell Nanoparticles for Magnetic Hyperthermia-Enhanced Catalysis

**DOI:** 10.1021/acsanm.6c01226

**Published:** 2026-04-24

**Authors:** Nahuel Nuñez, Carlos Díaz-Ufano, Alvaro Gallo-Cordova, Francisco Javier Palomares, María del Puerto Morales, Elin L. Winkler

**Affiliations:** † Instituto de Nanociencia y Nanotecnología (CNEA-CONICET), Av. Bustillo 9500, (8400) S. C. de Bariloche (RN), Argentina; ‡ Departamento Magnetismo y Materiales Magnéticos, Gerencia de Física, Centro Atómico Bariloche, Av. Bustillo 9500, (8400) S. C. de Bariloche (RN), Argentina; § 42670 Instituto Balseiro, CNEA-UNCuyo, Av. Bustillo 9500, (8400) S. C. de Bariloche (RN), Argentina; ∥ Escuela de Doctorado UAM, Centro de Estudios de Posgrado, Universidad Autónoma de Madrid. C/Francisco Tomás y Valiente, 2, 28049 Madrid, Spain; ⊥ 69570 Instituto de Ciencia de Materiales de Madrid, ICMM/CSIC, C/Sor Juana Inés de la Cruz 3, 28049 Madrid, Spain

**Keywords:** core@shell nanocatalysts, heterogeneous Fenton, magnetic hyperthermia, zinc ferrite, Cu-based catalyst

## Abstract

Core@shell nanocatalysts that integrate magnetic and catalytic functionalities provide a versatile platform for remotely controlled reactions. Here, we report Zn_0.2_Fe_2.8_O_4_ nanoparticles coated with a Cu­(II)-based shell, in which the zinc ferrite core generates heat under alternating magnetic fields, i.e., magnetic hyperthermia, while the copper shell exhibits heterogeneous Fenton-like activity. The ∼20 nm core shows high saturation magnetization (76 (4) emu/g) and superparamagnetic behavior at room temperature. Surface coating with an amorphous Cu­(II)-based phase is confirmed by XPS, EDS mapping, and ICP analysis. While bare Zn-ferrite nanoparticles show negligible Fenton-like activity, the copper-coated nanoparticles catalyze ·OH radical generation, as demonstrated by EPR experiments. The catalytic activity of the core@shell system remains low at room temperature but is strongly enhanced in magnetic hyperthermia conditions. Dye degradation assays using 100 ppm methylene blue and 1 mg/mL catalyst under alternating magnetic fields reveal that the core@shell system achieves 99% dye degradation in 1 h, exceeding the performance obtained under conventional thermal heating at equivalent bulk temperature. This enhanced performance could be attributed to a localized heating mechanism, in which the magnetic core acts as an internal heat source under alternating magnetic fields, promoting the heterogeneous redox reaction at the shell phase. These results demonstrate that the core@shell nanocatalysts with negligible activity at room conditions can be remotely enhanced by an alternating magnetic field, providing a promising strategy for applications where controlled catalytic activity is required.

## Graphical abstract

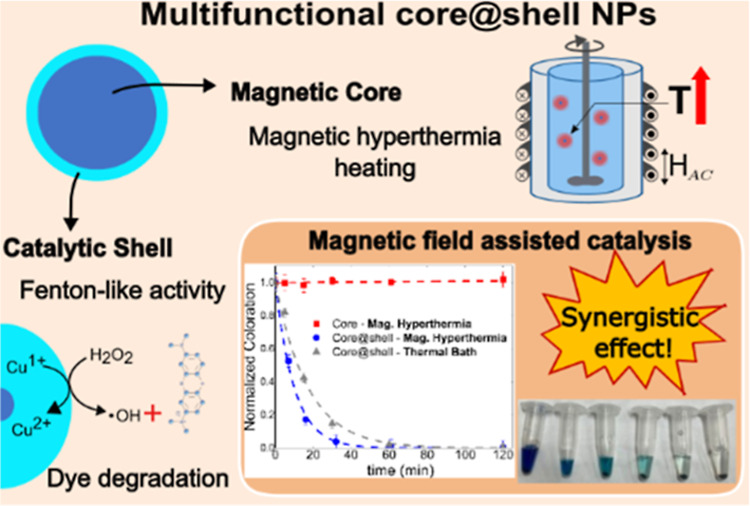

## 1 Introduction

Fenton-like catalysts have gained increasing attention in environmental^1–4^ and biomedical applications^5–9^ due to their ability to generate highly reactive oxygen species (ROS) under mild conditions. In this chemical process, a metal ion catalyzes the decomposition of hydrogen peroxide (H_2_O_2_), generating hydroxyl (·OH) and hydroperoxyl (·OOH) radicals. These radicals can oxidize organic molecules, leading to the mineralization of organic contaminants to remediate effluents, as well as induce oxidative stress in cellular environments, with this mechanism being the base of the development of innovative biomedical therapies.^10,11^

To enhance the catalytic activity, current research focuses on the design of advanced materials with multiple functionalities.^12,13^ As the Fenton reaction is a thermally activated process, the reaction kinetics can be exponentially enhanced by increasing the temperature. A promising strategy is the design of magnetic nanocatalysts with tailored magnetic properties that enable localized heating under alternating magnetic fields, i.e., magnetic hyperthermia (MH).^14^ By combining chemical activity with tunable magnetic properties, the efficiency of the materials can substantially improve.^15^ This synergistic effect is especially relevant in biomedical applications, where MH can be remotely and locally induced by turning on an AC magnetic field. This enhances the ROS production, thereby intensifying oxidative stress in the tumor microenvironment, leading to effective tumor regression or inhibition in animal models.^16,17^ Beyond medicine, MH has also been applied to trigger other catalytic reactions, such as CO_2_ hydrogenation for ammonia production^18^ or ammonia decomposition for hydrogen generation, highlighting the broader potential of this technology.^19^ In environmental remediation, magnetic nanomaterials also offer the unique advantage of magnetic harvesting, facilitating their reutilization in consecutive treatment cycles and improving the overall efficiency of wastewater treatment.

While temperature is a powerful tool to enhance the catalytic efficiency, the chemical stability of the selected material at elevated temperatures is critical for sustaining the reaction. In iron oxide nanoparticles (NPs), it is well established that Fe^2+^ ion is the active species with the highest kinetic rate in heterogeneous Fenton reactions.^20,21^ However, Fe^2+^ is highly prone to oxidation to Fe^3+^, a process that is further accelerated at higher temperatures. As a result, the catalytic efficiency of magnetite decreases upon oxidation, limiting its performance. To overcome this limitation, alternative metal ions, with both chemical activity and thermal stability, should be considered. One promising candidate is the copper metal ion, which has demonstrated the ability to maintain and even enhance its catalytic activity when temperature increases.^22^ However, although copper oxide is a stable phase, it is paramagnetic at room temperature, making it unsuitable for heating via MH.

One strategy to address this situation involves the design of advanced nanostructures combining different materials with a core–shell structure into a single nanoparticle. This architecture enables the independent optimization of the magnetic properties of the core and the catalytic activity of the shell, providing a versatile platform to improve the overall performance of the material.^23^ Similar approaches have been applied in systems like Fe_3_O_4_@CuO nanocomposite with photocatalytic activity to degrade organic dyes and with magnetic properties for magnetic recollecting.^24^ Also, carbon-encapsulated cobalt ferrite CoFe_2_O_4_/CoFe@C nanoparticles have been fabricated for the degradation of bisphenol.^25^ Another example includes the combination of an electrocatalytic shell (CoFe_2_O_4_) with a conductive core (Fe_3_O_4_) for water hydrolysis for renewable energy storage.^26^

In this context, we have developed a core@shell nanocatalyst, where the magnetic properties of the core were tailored to induce MH, while the shell exhibited Fenton-like catalytic activity with a good response under high-temperature conditions. Specifically, we synthesized Zn_0.2_Fe_2.8_O_4_ nanoparticles coated with a Cu­(II)-based shell for MH-enhanced catalytic degradation of organic dyes. In previous work, we optimized the stoichiometry and size of zinc ferrite nanoparticles to maximize the MH, achieving high magnetic saturation (M_S_) and tuning the magnetic relaxation by the Néel process.^27^ This mechanism is critical for the effective response of the nanoheater, regardless of the medium’s viscosity or the nanoparticle agglomeration. These optimized nanoparticles were then coated with a uniform copper hydroxide shell, followed by a thermal oxidation to promote the formation of CuO, yielding the core@shell architecture.

The Fenton-like catalytic activity of the core@shell NPs was evaluated by electron paramagnetic resonance (EPR) spectroscopy using the DMPO spin trap molecule, which enabled the identification and quantification of free radical species generated during the reaction. Moreover, the efficiency of the materials to degrade methylene blue (MB) was investigated under different conditions, such as the reaction temperature and heating method. Our results demonstrate that the catalytic performance of the Zn_0.2_Fe_2.8_O_4_@Cu­(II)-based nanocatalysts can be tuned by the copper content and operating temperature. This behavior is emphasized by the negligible chemical activity exhibited by the bare Zn_0.2_Fe_2.8_O_4_ nanoparticles. Notable, the MH significantly enhances the dye degradation, suggesting a synergistic effect between localized heating and heterogeneous catalytic activity. These findings highlight the potential of core@shell magnetic nanocatalysts as controllable and efficient Fenton-like systems for environmental remediation and targeted biomedical applications.

## 2 Experimental Section

### 2.1 Catalysts Synthesis

The chemical reagents used for the core NP synthesis are Fe­(III) acetylacetonate (97%, Sigma-Aldrich), Zn­(II) acetylacetonate (99.995%, Sigma-Aldrich), benzyl ether (98%, Sigma-Aldrich), 1,2-octanediol (98%, Sigma-Aldrich), oleic acid (99%, Sigma-Aldrich), and oleylamine (70%, Oleylamine). For the copper phase synthesis, the reagents used are CuCl_2_.2H_2_O (99%, Anedra) and NaOH.

Zinc ferrite nanoparticles, Zn_*x*_Fe_3‑x_O_4_, with nominal composition *x* = 0.4, were synthesized by high temperature decomposition, following the method described elsewhere.^27^ Briefly, 0.9222 g of Fe­(III) acetylacetonate and 0.1099 g of Zn­(II) acetylacetonate were mixed in 60 mL of benzyl ether using the surfactants 0.9 mL of oleylamine, 2.9 mL of oleic acid, and 0.24 mL of 1,2-octanediol. The solution was heated to 105 °C under N_2_ flow (0.1 mL/min) and intense mechanical stirring for 60 min. The mixture was then heated to 200 °C for 60 min and subsequently brought to reflux conditions (∼260 °C) for an additional 60 min. The synthesized material was washed using a solution of acetone and ethyl alcohol, followed by magnetic separation, which was repeated several times. The as-prepared nanoparticles were hydrophobic due to the oleic acid coating. To remove the oleic acid coating and enable the growth of the copper phase, the nanoparticles were washed with hot acetone (40 °C) for 48 h, with intermittent ultrasonication. This procedure follows a protocol previously reported by our group (see Supporting Information of ref 28). After this treatment, the nanoparticles become readily dispersible in polar solvents, such as water and alcohol, indicating the successful removal of the hydrophobic surfactant layer.

Once the oleic acid was removed from the nanoparticles, the copper phase was precipitated onto them. For this purpose, two solutions were prepared: one containing 50 mg of nanoparticles in 50 mL of ethanol and the other containing 20 mg of copper salt (CuCl_2_·2H_2_O) in 20 mL of ethanol. After ultrasonic dispersion, the nanoparticle solution was maintained under constant ultrasonication, and the copper solution was gradually added. Mechanical stirring was continued for 1 h to promote copper adsorption onto the nanoparticle surface. Subsequently, a solution of 20 mg of NaOH in 20 mL of ethanol was added dropwise. The addition of the base induced the precipitation of copper as copper hydroxide. The resulting suspension was stirred for 24 h and washed three times with ethanol, followed by magnetic separation. The obtained material was resuspended in 50 mL of triethylene glycol and heated to 210 °C for 2 h to convert copper hydroxide into copper oxide. Finally, the material was repeatedly washed with ethanol, separated magnetically, and dried at 50 °C. The sample was named CS1. To evaluate the control of copper coating on the nanoparticles, this procedure was repeated with another batch of nanoparticles but using a lower copper content. In this case, the copper solution was prepared by dissolving 14 mg of CuCl_2_·2H_2_O in 14 mL of ethanol. Similarly, 14 mg of NaOH in 14 mL of ethanol was used to precipitate the hydroxide. The rest of the procedure followed the same protocol as that described above. The sample is called CS2.

### 2.2 Characterization Techniques

The crystal structure of the synthesized samples was characterized using X-ray diffraction (XRD) on a Bruker Advance D8 diffractometer (Cu Kα radiation, λ = 0.15406 nm). The concentration of iron, zinc and copper ions into the nanoparticle structure was determined by inductively coupled plasma optical emission spectroscopy (ICP-OES) using a PerkinElmer OPTIMA 2100 DV spectrometer.

X-ray photoelectron spectroscopy (XPS) has been used to characterize the elemental composition and the oxidation states of Fe, Zn, and Cu present on the surface of the samples. XPS experiments were performed in a UHV chamber with a base pressure of 10–10 mbar equipped with a hemispherical electron energy spectrometer (Phoibos 150, SPECS Surface Nano Analysis GmbH, Germany) and a 2D delay-line detector (Surface Concept GmbH, Germany), using an X-ray source of Al–Kα (1486.6 eV).^29^ XPS spectra were acquired at normal emission takeoff angle, using an energy step of 0.50 and 0.10 eV and a pass energy of 40 and 20 eV for survey spectra and detailed core level regions, respectively. The surface charging effect built up upon the photoemission experiments has been compensated by using a low-energy electron flood gun. The absolute binding energies of the photoelectron spectra were determined by referencing the C 1s photoelectron peak at 285.0 eV. Both the peak energy and line shape of the C 1s peak are checked prior to and after the measurement of every selected core-level transition. The spectra were analyzed with the CasaXPS program (Casa Software Ltd., Cheshire, UK) using a Shirley method for background subtraction and data processing for quantitative XPS analysis. Spectra are displayed after the subtraction of the contribution of the Al–Kα satellite emission. The Zn/Fe and Cu/Fe ratios were determined by measuring the integral peak areas for each element after background subtraction and normalization using sensitivity factors by the electron energy analyzer manufacturer.

Zeta potential measurements were performed by using a Malvern Zetasizer Nano SZ (Malvern, UK) equipped with a 633 nm He–Ne laser. The nanoparticle surface charge was evaluated as a function of the pH at room temperature. The pH of the suspensions was adjusted between 2 and 11 using HNO_3_ and KOH, employing 10^–2^ M KNO_3_ as a supporting electrolyte. These measurements were used to determine the isoelectric points of the samples.

The size and morphology of the nanocatalysts were analyzed by high-resolution transmission electron microscopy (HR-TEM) using a Philips CM-200 microscope operating at 200 kV. High-angle annular dark-field scanning transmission electron microscopy (HAADF–STEM) coupled with energy-dispersive X-ray spectroscopy (EDS) was performed on a JEM-2100 (JEOL) microscope to obtain spatial distribution maps of the transition metals (Fe, Cu, Zn). The DC magnetization of the samples was measured by using a LakeShore 7300 vibrating sample magnetometer (VSM). Magnetization versus applied field (M­(H)) curves were acquired at room temperature under an applied field of up to ± 10 kOe.

Magnetic hyperthermia measurements were performed to evaluate the heating performance under an alternating magnetic field. The specific absorption rate (SAR), which quantifies the power dissipated per unit mass of magnetic material, was determined according to

$$SAR=\left(C_{P}\frac{m_{solv}}{m_{NPs}}\right)\left(dT/dt\right)$$

where *C*_*p*_ is the specific heat capacity of the system, *m*_solv_ is the mass of the solution, *m*_NPs_ is the mass of magnetic material, and *dT*/*dt* is the initial slope of the temperature–time curve obtained by linear fitting of the first 4 min after switching on the alternating magnetic field, where the heating rate remains approximately constant. For aqueous dispersions at a low nanoparticle concentration, *C*_*p*_ can be approximated by the specific heat capacity of water (4185 J·L^–1^·K^–1^). Measurements were carried out using a FIVES CELES AMF induction system (Model 12118 M01, France) equipped with a water-cooled copper coil (50 mm diameter, six turns). Experiments were conducted at nanoparticles concentration of 1 mg/mL, with a magnetic field of 60 mT and 96 kHz. This combination corresponds to the minimum frequency allowed by the equipment, at which the maximum magnetic field amplitude can be applied. Such conditions were selected considering the nanoparticle size (∼20 nm and larger), since previous studies have shown that higher magnetic field amplitudes favor efficient heating in this size range.^30^ Temperature evolution was monitored in real time with a fiber optic sensor inserted into the sample. During the experiments, the dispersion was kept under continuous stirring to maintain a homogeneous suspension and prevent nanoparticle aggregation or sedimentation under alternating magnetic field conditions.

The generation of free radicals by the catalysts was investigated by using electron paramagnetic resonance (EPR) spectroscopy in the X-band (9.5 GHz) at room temperature. Measurements were performed with a BRUKER ELEXSYS II-E500 spectrometer, employing the nitrone-based spin trap 5,5-dimethyl-1-pyrroline *N*-oxide (DMPO). A modulation frequency of 100 kHz and 3 Oe was used. In these experiments, 0.1 mg/mL of catalyst was dispersed in 100 mM acetate buffer (pH = 5), followed by the addition of 25 μL of a 1 g:6 mL DMPO:DMSO solution. Subsequently, 5 μL of a 30% H_2_O_2_ aqueous solution was added. Approximately 90 μL of the reaction solution was contained in a quartz tube with the measured region having a height of 30 mm. A pattern sample of MgO/Mn^2+^ was attached to the quartz tube in order to acquire the EPR signal of Mn^2+^ simultaneously with the corresponding free radicals to normalize the production of each sample. The evolution of the reaction with time was followed by measuring the EPR spectra at intervals of no more than 10 min along 1 h each reaction. The EPR spectra were fitted using the Bruker SPIN program, based on an isotropic spin Hamiltonian including Zeeman and hyperfine interactions for each free radical DMPO adducted species. The spectra corresponding to the dominant ·OH radical (DMPO/·OH), characterized by four equidistant peaks with an intensity ratio of 1:2:2:1, were fitted considering the hyperfine interaction of the unpaired electron with the neighboring nitrogen nucleus, *a*_*N*_ ≈ 14.9 Oe, *I*_*N*_ = 1, and the hydrogen nucleus, *a*_*H*_ ≈ 14.8 Oe, *I*_*H*_ = 1/2, where a denotes the hyperfine coupling constant and I the nuclear spin. The signal intensity, obtained from the double integral of the fitted spectra, is proportional to the concentration of the radicals. Therefore, the temporal evolution of the radical concentration generated during the reaction was monitored by fitting the signal intensity as a function of time while keeping the spectroscopic parameters (*a*_*N*_, *a*_*H*_, and line width) constant.

To evaluate the capability of the nanocatalysts to degrade organic compounds, colorimetric experiments were conducted using methylene blue dye. The experimental conditions were selected within the typical ranges reported for heterogeneous Fenton and Fenton-like processes in order to allow a comparison with previous studies. In these experiments, the catalyst was dispersed in acetate buffer (pH = 5) at a concentration of 1 mg/mL, while the dye concentration was set to 100 ppm. A pH of 5 was chosen as a compromise between promoting Fenton-like reactions and avoiding excessive metal leaching at lower pH values.^22^

Temperature control was achieved using a thermal bath (TB) set to 20, 53, or 60 °C. The system was kept under continuous agitation for 2 h to ensure adsorption of the dye onto the nanoparticle surface. Subsequently, 10 μL/mL of a 30% H_2_O_2_ aqueous solution was added, and the degradation efficiency was determined by measuring the absorbance at 663 nm at different time intervals (5, 15, 30, 60, and 120 min). Colorimetric measurements were performed with a NUMAK 721 UV–vis spectrophotometer.

To quantitatively compare the catalytic performance under different conditions, the kinetic data were analyzed by using a pseudo-first-order (PFO) model. The apparent rate constants (*k*) were obtained from the linear fitting of -ln­(*C*/*C*_0_) as a function of time, where *C*_0_ and *C* correspond to the dye concentration at *t* = 0 and time *t*, respectively.

To evaluate catalyst recyclability, consecutive degradation cycles were performed under thermal bath conditions at 60 °C. After each cycle, the nanoparticles were magnetically separated from the reaction medium, washed with deionized water, and dried overnight before being reused under identical experimental conditions. The same procedure for methylene blue degradation described above was followed in each cycle.

Finally, the methylene blue degradation process was investigated under magnetic hyperthermia conditions. In this case, an alternating magnetic field of 60 mT and 96 kHz was applied in the absence of the thermal bath and with constant stirring, while the rest of the protocol remained the same as described above.

## 3 Results and Discussion

Core@shell magnetic nanocatalysts were obtained in a two-step synthesis process, as illustrated in Scheme 1. First, zinc ferrite magnetic nanoparticles were synthesized following a protocol previously reported,^27^ using the method of high-temperature decomposition of organic salt precursors. In a second step, the precipitation of copper hydroxide onto the nanoparticles surface was carried out.^31^ Here, the nanoparticles were dispersed in ethanol, and then copper chloride was added to the solution with an atomic ratio $\frac{Cu}{Fe}=0.18$ The solution was maintained under ultrasound for 20 min while NaOH was added to precipitate the copper ions in the form of hydroxide on the ferrite nanoparticle surface. The product was repeatedly washed in ethanol and redispersed in triethylene glycol, and then the solution was heated to 210 °C to transform copper hydroxide into copper oxide. The sample was named CS1. Finally, the nanoparticles were magnetically precipitated and washed with ethanol, then dried, and stored as powder. For comparison, nanoparticles with reduced copper chloride content, that is, $\frac{Cu}{Fe}=0.13$ atomic ratio, were prepared in order to follow the Cu-dependent behavior; in this case, the sample was named as CS2. See the experimental section for more details about the synthesis process.

**Scheme 1:**
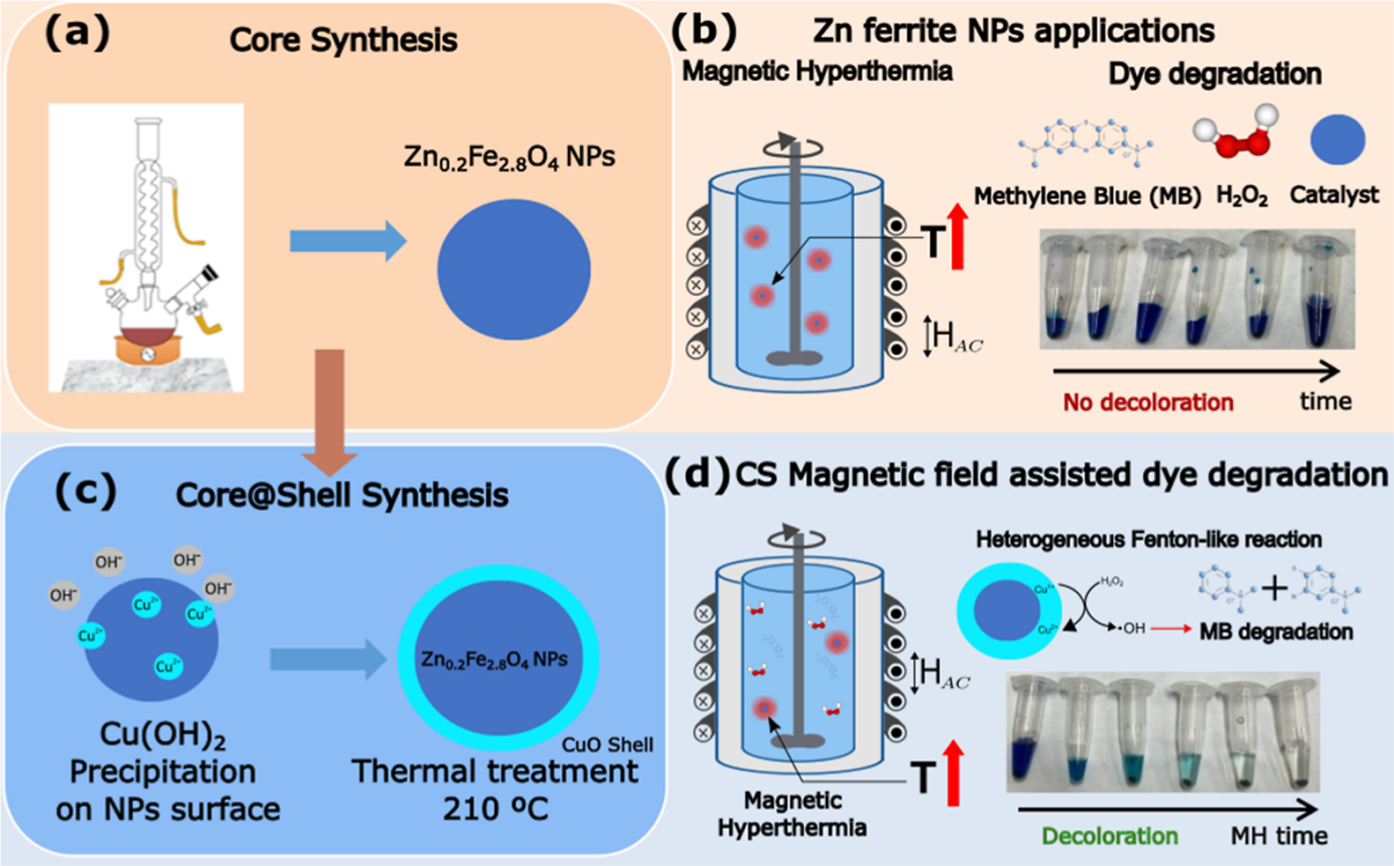
Schematic Illustration of Nanoparticle Fabrication and Applications. (a) Synthesis of Zn_0.2_Fe_2.8_O_4_ Nanoparticles via the High-Temperature Decomposition of Organic Precursors, and (b) Their Application for Magnetic Hyperthermia and Dye Degradation. (c) Synthesis of Zn_0.2_Fe_2.8_O_4_@Cu­(II)-Based core@shell Nanoparticles by Precipitation of a Copper Hydroxide Onto Zinc Ferrite Nanoparticles and (d) Their Application in Magnetic Hyperthermia and Dye Degradation

### 3.1 Nanocatalysts Characterization

The structural characterization of synthesized materials was performed by using X-ray diffraction (XRD) and transmission electron microscopy (TEM). XRD analysis of the core nanoparticles confirmed the *Fd*3̅*m* cubic spinel structure, as evidenced by well-defined diffraction peaks (Figure 1a). The *a* lattice parameter was estimated to be 0.842 nm, which is slightly larger than that of pure magnetite (0.839 nm) and closer to that of zinc ferrite 0.844 nm.^32,33^ This increase is consistent with the larger ionic radius of Zn^2+^ compared to Fe^3+^, where Zn^2+^ preferentially occupied the tetrahedral site in the spinel.^34^ The crystallite size, estimated by using the Scherrer equation, was determined to be 22 nm. The XRD pattern of both CS1 and CS2 core@shell systems did not exhibit detectable peaks associated with copper phases, suggesting that the deposited copper is either amorphous or forms a layer too thin to be detected by the XRD diffraction pattern.

**Figure 1:**
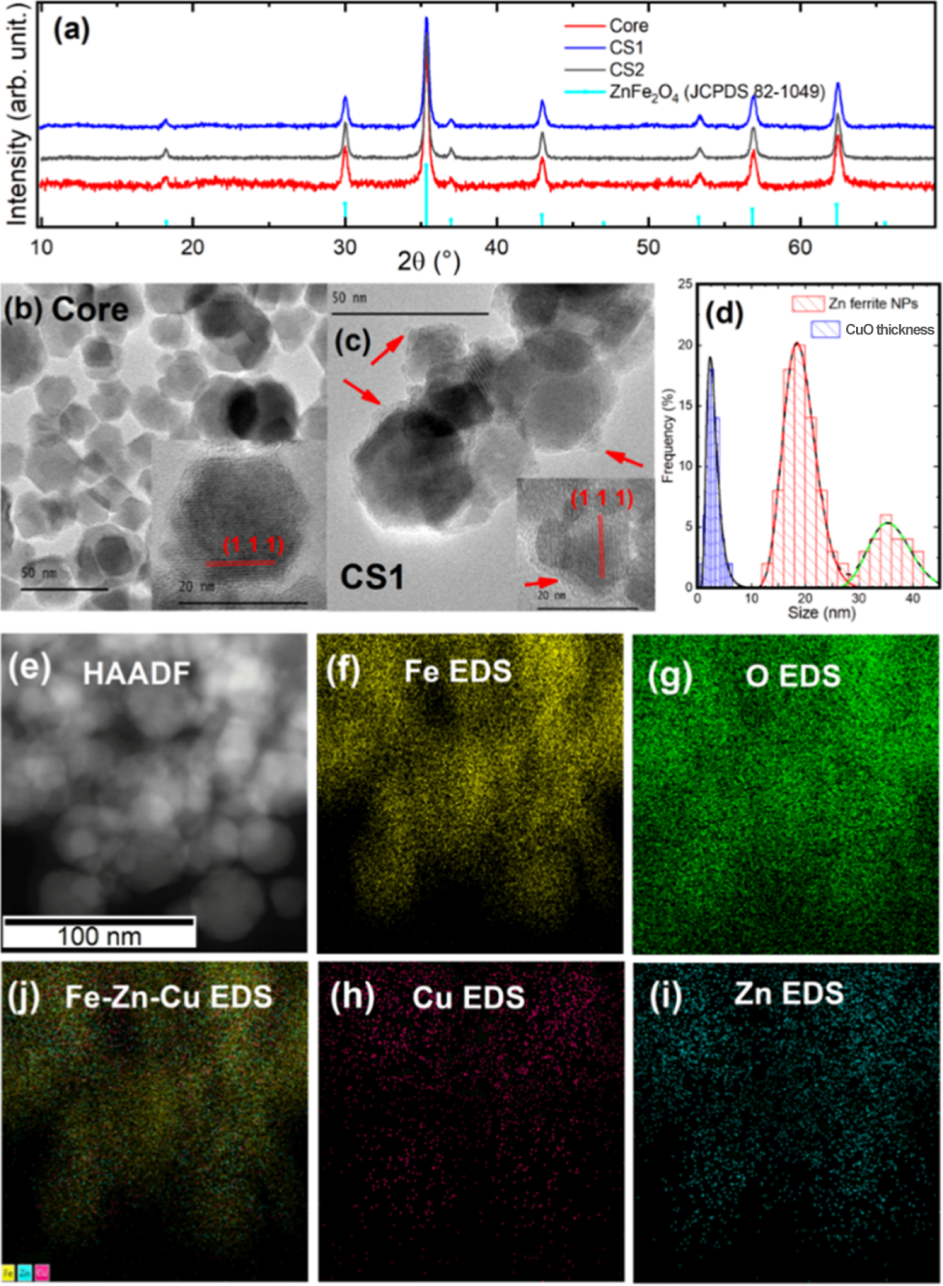
Structural and compositional map characterization of core and core@shell systems. (a) X-ray diffractograms of core zinc ferrite (red), core@shell CS1 (blue), and CS2 (black) nanoparticle systems. Reference peaks corresponding to the cubic phase of ZnFe_2_O_4_ with the Fd3m space group and the standard diffraction pattern JCPDS card no: 01-082-1049,^35^ are also shown. TEM images of core (b) and CS1 (c) systems; the insets show high-resolution micrographs where the (111) planes are indexed. The red arrows indicate amorphous copper oxide precipitates on the surface of the zinc ferrite nanoparticles. (d) Size distribution of the zinc ferrite nanoparticles (red) and of the copper phase coating thickness (blue) in the CS1 system; the log–normal fitting curves are shown. (e) HAADF image of the CS1 NPs. STEM compositional mapping of: (f) Fe, (g) O, (h) Cu, and (i) Zn. The combined STEM compositional mapping of Fe, Cu, and Zn is presented in (j).

TEM image analysis shows that the core nanoparticles exhibited high crystallinity, as evidenced by the high-resolution image inset of Figure 1b, where well-defined (111) crystalline planes were identified. The size distribution analysis indicates that the zinc ferrite system has a bimodal distribution, as shown in Figure 1d. The size distribution was fitted with two log–normal distributions, showing a primary peak at 19 ± 3 nm and a secondary peak at 35 ± 4 nm, which may indicate the early stages of particle sintering.

TEM analysis of the CS1 NPs is presented in Figure 1c. An irregular and amorphous coating on the nanoparticle surfaces is clearly observed, likely due to copper oxide deposition; however, the presence of Cu­(OH)_2_ cannot be ruled out. The average thickness of these shell structures is 2.8 ± 0.9 nm (Figure 1d). The high-resolution images of Figure 1c further revealed highly crystalline zinc ferrite nanoparticles sharing crystal planes, suggesting early-stage sintering coated with an amorphous layer.

To further investigate the elemental composition map of the nanoparticles, high-angle annular dark field (HAADF) imaging combined with energy-dispersive X-ray spectroscopy (EDS) was employed. Figure 1e shows the HAADF image of the CS1 NPs, while Figure 1f–i displays the EDS elemental mappings for Fe, O, Cu, and Zn, respectively. The integrated elemental distribution is shown in Figure 1j. These measurements confirm the spatial colocalization of iron, zinc, and copper in the core@shell architecture. The oxygen map reveals that, while the highest oxygen signal corresponds to the nanoparticle’s regions, residual oxygen is also present in areas without nanoparticles, which can be attributed to residual organic material in the sample. The HAADF–EDS measurements of CS2 NPs are shown in Figure S1, which further support the successful coating of the Zinc ferrite NPs with the copper phase.

The overall composition of the nanoparticles was determined by ICP spectrometry, and the results are presented in Table 1. The Zn/Fe ratio was measured in the uncoated nanoparticles, resulting in Zn/Fe ∼0.07, corresponding to a Zn_0.2_Fe_2.8_O_4_ core stoichiometry. The Zn incorporation in the spinel structure is considerably lower than the nominal composition, as it is usually observed in nanoparticle systems synthesized by high-temperature decomposition of organic precursors.^33,36^ In the core@shell nanoparticles, the atomic Cu/Fe ratio results in 0.15 and 0.09 for the CS1 and CS2 samples, respectively; indicating that the shell concentration can be modified by the amount of Cu salt added during the second step of the synthesis.

**Table 1:** Nominal Zn/Fe and Cu/Fe Atomic Ratios and the Corresponding Values Obtained from ICP and XPS Analysis for the Core and Core/Shell Samples as-prepared (Core, CS1, and CS2) and After One Catalytic Cycle (used), with the Later Determined from Zn, Fe, and Cu 3p core-level XPS Emissions

	nominal atomic ratio		ICP atomic ratio		XPS				
sample	Zn/Fe	Cu/Fe	Zn/Fe	Cu/Fe	3p relative intensity			atomic ratio	
					Zn	Fe	Cu	Zn/Fe	Cu/Fe
Core	0.16	-	0.07	–	30.7	69.3	–	0.44	–
CS1	0.16	0.18	0.05	0.15	13.9	59.3	26.3	0.23	0.44
CS2	0.16	0.13	0.06	0.09	–	–	–	–	–
Core (used)	-	-	0.04	–	7.9	92.1	–	0.08	–
CS1 (used)	-	-	0.04	0.12	6.8	78.0	15.2	0.09	0.19

To gain further insight into the surface composition, X-ray photoelectron spectroscopy (XPS) measurements were performed on core and CS1 systems. Figure 2 shows the high-resolution spectra of core and CS1 samples in the 50–100 eV binding energy region, where the 3p peaks of Fe, Zn, and Cu are clearly observed. Table 1 summarizes the relative atomic percentages and the Zn/Fe and Cu/Fe ratios derived from the 3p core-level regions. For the Zn_0_._2_Fe_2_._8_O_4_ core sample, the atomic Zn/Fe ratio (0.44) is markedly larger than that determined by ICP, indicating a partial Zn enrichment at the surface compared with the bulk composition. After coating with copper phase (CS1), the Cu signal becomes clearly evident in the 3p region, corresponding to ∼26 at. % Cu at the surface. The higher Cu/Fe ratio derived from the 3p levels (0.44) compared with that obtained by ICP (0.15) confirms the localization of the Cu species in the outermost layer of the nanoparticles, in the form of Cu­(II), most likely corresponding to CuO, although the presence of Cu­(OH)_2_ cannot be ruled out. Simultaneously, the Zn/Fe ratio decreases (to 0.23 from the 3p region), suggesting some Zn leaching during the copper hydroxide precipitation step.

**Figure 2:**
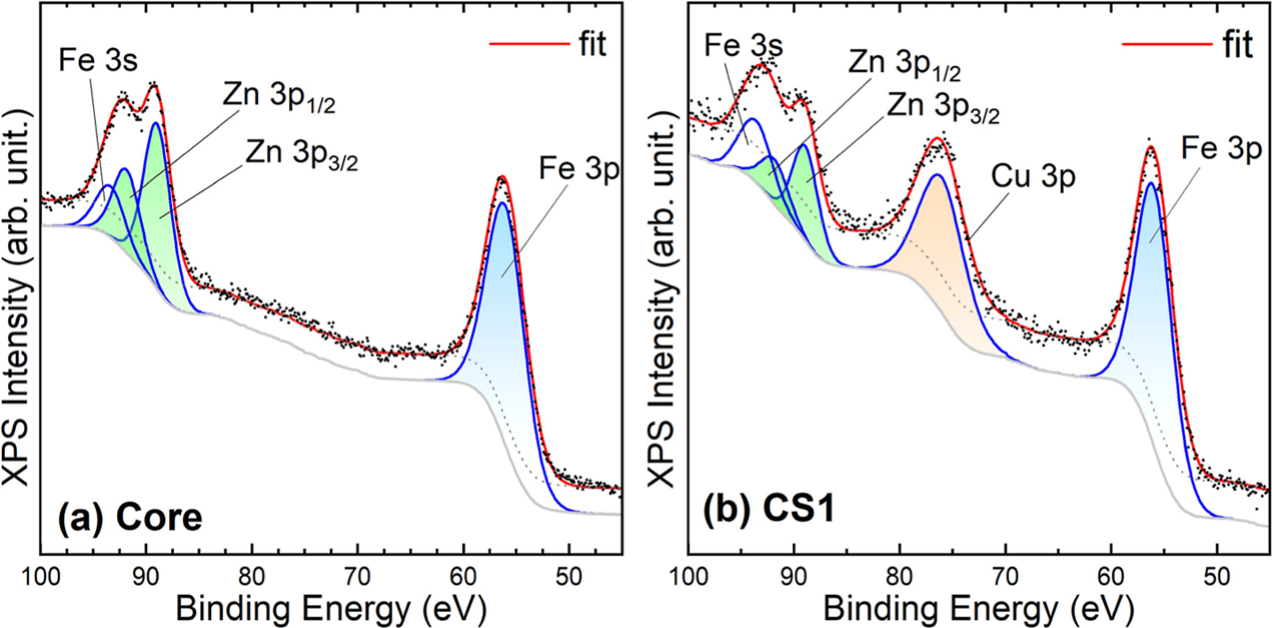
High-resolution XPS spectra of (a) Zn_0.2_Fe_2.8_O_4_ core and (b) Zn_0.2_Fe_2.8_O_4_@Cu­(II)-base CS1 nanoparticles in the 50–100 eV binding energy region, highlighting the Fe, Zn, and Cu 3p core-level peaks. The spectra evidence the emergence of Cu signals in CS1, consistent with the formation of a CuO/Cu­(OH)_2_ shell.

The surface charge of the nanoparticles was further evaluated through *Z*-potential measurements as a function of pH (Figure S2). The bare Zn_0.2_Fe_2.8_O_4_ nanoparticles show an isoelectric point around pH ≈6.5, while the core@shell system CS1 exhibits a shift toward higher pH values (≈8.5), consistent with the presence of a copper oxide coating on the nanoparticle surface.

Magnetic characterization was carried out by using a vibrating sample magnetometer (VSM). Figure 3a shows the field dependence of the magnetization for both the core and core@shell systems measured at room temperature, including an inset displaying the low-field hysteresis loop. The M­(H) curves exhibit a nearly reversible behavior characteristic of the superparamagnetic regime. It is well established that Zn substitution in ferrites reduces the magnetic anisotropy;^37–39^ combined with the nanometric particle size, this accounts for the observed superparamagnetic behavior in spite of the relatively large particle size (>15 nm). From the M­(H) curves, the saturation magnetization of the Zn-ferrite core nanoparticles was determined as *M*_*S*_ = 76(4) emu/g, a value close to bulk magnetite (84–88 emu/g).^40–42^ Small amounts of Zn ions doping in the magnetite lattice ([Fe^3+^]_A_[Fe^2+^Fe^3+^]_B_O^2–^_4_) have been reported to enhance magnetization despite the diamagnetic nature of Zn^2+^.^43^ This is because Zn^2+^ ions tend to replace Fe^3+^ in the A site, reinforcing the already existing imbalance between antiferromagnetically coupled A and B sublattices.^44^ This may account for the high magnetization observed here despite the surface disorder typically expected at the nanoscale, which usually reduces magnetization. In the core@shell systems, the magnetization decreases significantly to 61(4) emu/g for CS1 and 66(3) emu/g for CS2, due to the lower proportion of magnetic material in the overall structure. The remanent magnetization (*M*_*r*_) and coercive field (*H*_*C*_) values are summarized in Table S1. The low *M*_*r*_/*M*_*s*_ ratios (∼0.05-0.08) indicate a predominantly superparamagnetic behavior, although not ideal, suggesting the presence of a small fraction of magnetically blocked particles, likely associated with larger particles within the size distribution. Consistently, the low coercive fields (*H*_*C*_ ≈ 20–30 Oe) confirm a magnetically soft behavior, in agreement with the predominant superparamagnetic regime observed at room temperature.

**Figure 3:**
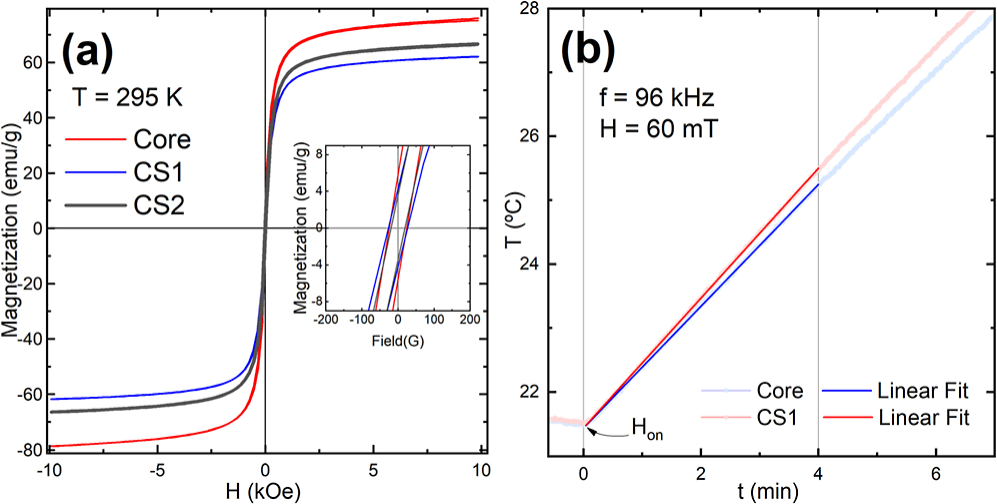
(a) Field-dependent magnetization curves of Zn_0.2_Fe_2.8_O_4_ (core) and Zn_0.2_Fe_2.8_O_4_@Cu­(II)-based (core@shell) nanoparticles at room temperature showing nearly reversible behavior, consistent with a superparamagnetic regime; the inset displays the low-field hysteresis loop. (b) Magnetic hyperthermia measurements of core and CS1 samples under an alternating magnetic field (96 kHz, 60 mT) at 1 mg/mL concentration in water; the time window used for SAR determination and the corresponding linear fit are also indicated.

To evaluate the AC response of the nanoparticles, magnetic hyperthermia measurements were performed under an alternating magnetic field of 96 kHz and 60 mT, with the nanoparticles dispersed in water at a concentration of 1 mg of NPs/mL. Figure 3b shows the temperature increase over time for the core and CS1 systems. The heating rate, obtained from the initial slope of the temperature vs time curve, was ∼0.017 K/s for both systems. Using the specific heat capacity of water (*C*_*P*_ = 4.18 J g^–1^ K^–1^) and accounting for the mass of solvent and nanoparticles ( $\frac{m_{NPs}}{m_{solv}}$ = 0.001), the specific absorption rate (SAR) was calculated to be ∼70 W/g (see experimental section to further details). This value is in line with previously reported SARs for zinc ferrite-based nanoparticles of comparable size and composition.^45^

Overall, the structural and magnetic characterization confirms the successful synthesis of core@shell nanostructures consisting of a zinc ferrite core with favorable magnetic properties and a copper oxide/hydroxide shell that is expected to have catalytic functionality. Under an alternating magnetic field, the nanoparticles efficiently convert electromagnetic energy into heat, which is particularly advantageous for degradation processes as the elevated local temperature accelerates Fenton-like catalytic reactions at the copper shell.

### 3.2 EPR Analysis of Free Radical Generation

The generation of free radicals by the core and the core@shell NPs during the heterogeneous Fenton-like reaction was evaluated by using EPR spectroscopy. Due to the high reactivity and short lifetime of these radical species, direct detection is not feasible. To overcome this limitation, the spin-trapping technique was used, which implements diamagnetic molecules known as spin traps that react with the free radicals to form more stable radical adducts that can be detected by EPR. In particular, the DMPO spin trap used in this study produces different EPR spectra depending on the trapped species, allowing identification of the reactive radicals involved and providing insight into the reaction mechanism and kinetics.^21^

Figure 4a displays representative EPR spectra of the reaction system containing uncoated Zn_0.2_Fe_2.8_O_4_ and CS1 Zn_0.2_Fe_2.8_O_4_@Cu­(II)-based NPs, measured at room temperature. The reaction solution contains the NPs dispersed in acetate buffer at pH 5 in the presence of H_2_O_2_ and the DMPO spin trap. The spectra shown in Figure 4a were recorded 10 min after the addition of H_2_O_2_, which initiated the catalytic reaction. For comparison, the figure also includes the spectrum of the blank solution measured under identical conditions but without nanoparticles. It is important to note that the area under the EPR absorption spectra (determined by double integration of the resonance line) is proportional to the concentration of the paramagnetic species.^28^ In order to ensure a reliable comparison between samples, all EPR signals were normalized using the spectrum of a MgO/Mn^2+^ crystal, measured simultaneously during the reaction by attaching the crystals to the same EPR quartz tube.

**Figure 4:**
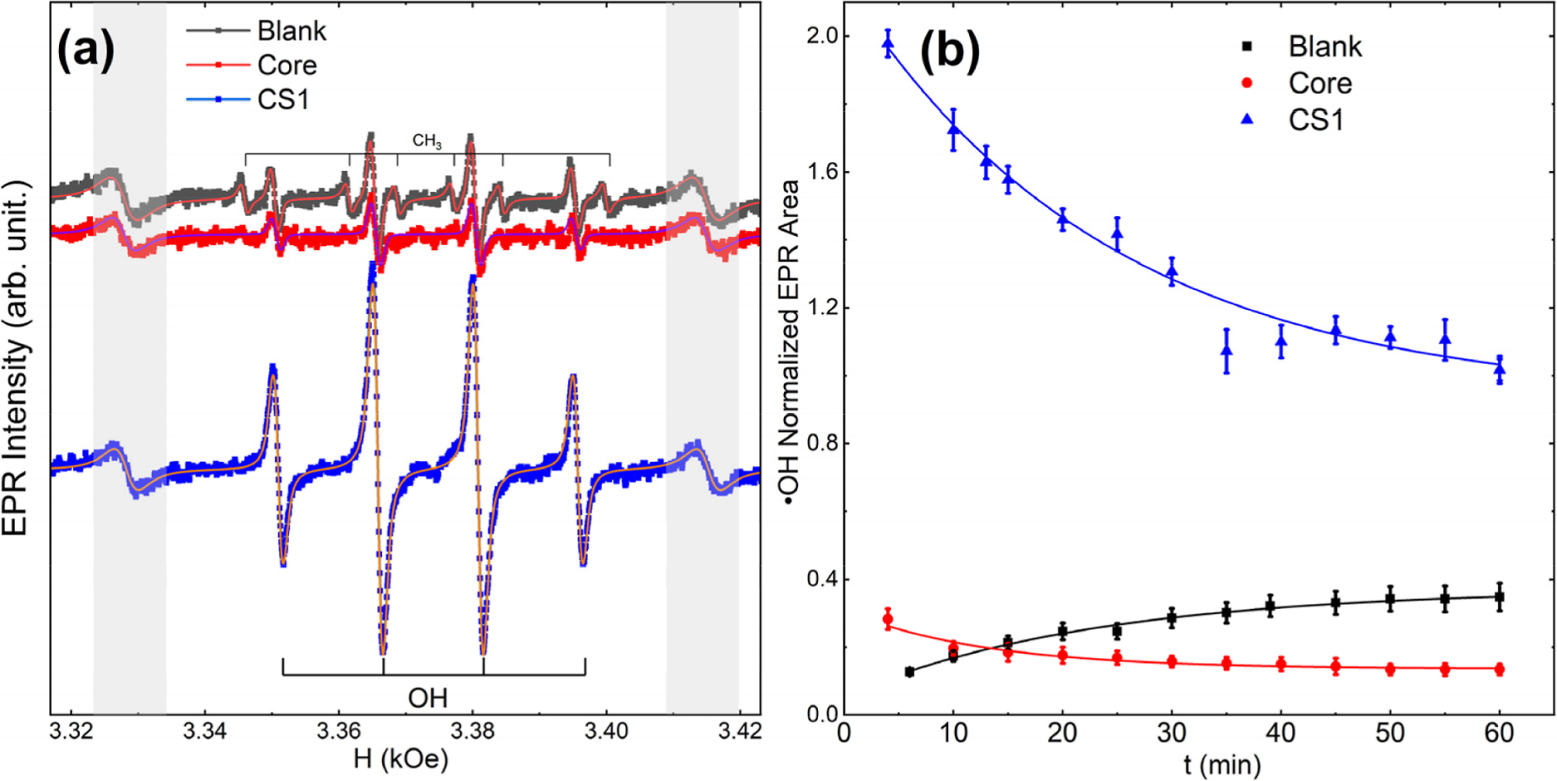
(a) EPR spectra and the corresponding fitting curve of the free radicals generated by the core (red) and CS1 core@shell systems (blue) and the blank solution without nanoparticles (black). The spectra were recorded 10 min after the reaction started, using DMPO as a spin trap. The shadow regions signal the resonance of the Mn^2+^ from the pattern sample. (b) Kinetics curves of ·OH radical production for the different reaction solutions using different catalysts; the lines are a guide to the eye.

From Figure 4a, it is notable that the EPR intensity in the solution containing the core zinc ferrite nanoparticles is similar to the blank sample, while it increases significantly when the reaction is catalyzed by the core@shell NPs. The resonance lines were fitted with the hyperfine parameters reported for the free radical-DMPO adducts by the Spin Trap Database of the National Institute of Environmental Health Sciences.^46^ The main signal observed in all of the reaction solutions corresponds to the ·OH radical resonance, characterized by four equidistant peaks with an intensity ratio of 1:2:2:1. Additionally, in the blank solution a small resonance signal attributed to the ·CH_3_ radical can be recognized; however, its intensity was very low, and it did not appear in the other experiments, so it was excluded from the analysis.

The kinetics of the reaction was followed by recording the spectrum along 60 min of reaction. Figure 4b presents the time dependence of the ·OH EPR resonance area obtained by fitting the EPR signal and normalizing it with the area of the Mn^2+^ reference signal. The first remarkable observation is that, after 10 min of reaction, the concentration of free radicals measured in the zinc ferrite (core) NPs is lower than that observed in the blank solution. Considering that Fe^2+^ and Fe^3+^ ions react with hydrogen peroxide, generating ·OH and ·OOH radicals, respectively, the observed inhibition of free radicals is attributed to the presence of Zn in the nanoparticles. This phenomenon has been previously attributed to the redox inactivity of the Zn^2+^ due to the stability of its 3d^10^ electron configuration, which passivates active sites and suppresses the redox reactions that generate ROS.^39,47^ The second notable observation is the enhancement of the ·OH radical concentration, by an order of magnitude, when the reaction is catalyzed by the core@shell nanoparticles, which can be attributed to the participation of copper species in Fenton-like reactions through a Cu^2+^/Cu^+^ redox cycle, analogous to the classical Fe^3+^/Fe^2+^ mechanism. This comparison clearly evidences the active role of the copper phase coating in free radical production. Considering that the ·OH radical is the ROS with the highest oxidizing power, this result is highly promising for the intended application. Although the EPR signal provides a clear fingerprint of ·OH radical generation, it does not discriminate between radicals produced via homo- or heterogeneous Fenton pathways. This distinction is particularly relevant, since post-reaction analysis indicates partial Cu leaching, which may contribute to a homogeneous Fenton reaction; this effect will be further addressed through degradation experiments in the next sections.

### 3.3 Catalytic Activity versus Temperature

After confirming the catalytic activity of the core@shell samples to generate ·OH radicals, we evaluated their potential for degrading organic compounds at different temperatures. Methylene blue (MB) was selected as the model compound for these studies. Degradation experiments were performed at 20 and 60 °C in acetate buffer at pH 5, using a nanoparticle concentration of 1 mg/mL, 100 ppm of dye, and 10 μL/mL of H_2_O_2_.

Figure 5a presents the MB degradation after 2 h of reaction time using bare Zn_0.2_Fe_2.8_O_4_ nanoparticles and the two core@shell systems, CS1 and CS2. The results were compared with a control experiment, carried out in the absence of nanoparticles. The absorbance values at 663 nm were normalized to the absorbance measured at *t*_0_, defined as the time of H_2_O_2_ addition, which occurs after the 2 h period of methylene blue adsorption onto the nanoparticles. The bare zinc ferrite nanoparticles system exhibits negligible catalytic activity, whereas the core@shell systems effectively degrade the dye. These results are consistent with the EPR findings and support a correlation between the dye degradation and the ·OH radical generation.

**Figure 5:**
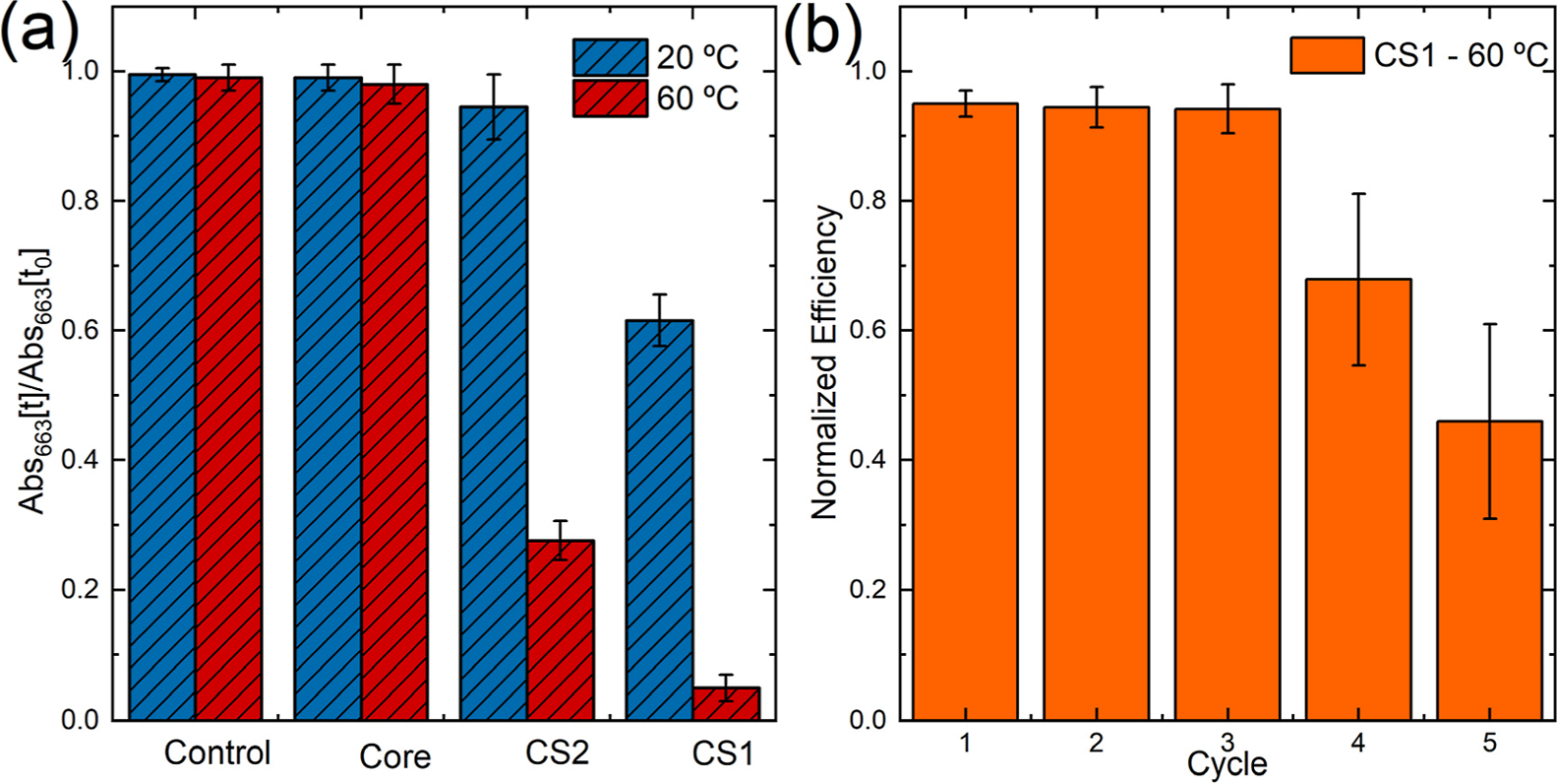
(a) Normalized degradation of methylene blue solutions using bare zinc ferrite nanoparticles and CS1 and CS2 core@shell systems after 2 h of reaction, measured from *t*_0_, at two working temperatures controlled by a thermal bath at 20 °C (blue bars) and 60 °C (red bars). Here, *t*_0_ corresponds to the time of H_2_O_2_ addition after a 2 h adsorption period. The experiments were performed using 1 mg/mL of catalyst, 100 ppm of dye, and 10 μL/mL of H_2_O_2_ in acetate buffer (pH 5). (b) Degradation efficiency of the methylene blue solution using the CS1 system at 60 °C over five successive cycles. Error bars represent the standard deviation calculated from at least three independent experiments. The degradation values were normalized to the absorbance measured at *t*_0_.

The degradation efficiency correlates with the amount of Cu loading. At room temperature CS2 shows only 5% of degradation, while CS1 achieves 40% after 2 h of reaction. As expected for a thermally activated process, the degradation improves significantly above room temperature. For the CS2 sample, the efficiency increases from 5% to 75% at 60 °C, while, for CS1, nearly complete degradation of MB is achieved at 60 °C.

In addition, the recyclability of the CS1 catalyst was evaluated through consecutive cycles at 60 °C. After each reaction, the nanoparticles were magnetically separated, dried overnight, and reused under identical conditions. As shown in Figure 5b, the catalyst maintains a high efficiency for at least three cycles, after which a gradual decrease in activity is observed.

These results demonstrate the activation of the nanoparticles through the zinc ferrite coating with Cu­(II)-phases and increased temperature, highlighting the core@shell system as a promising candidate for thermally controlled catalytic applications.

### 3.4 Magnetically Enhanced Catalytic Activity

In the previous sections, we demonstrated that the bare magnetic NPs present negligible catalytic activity in the measured temperature range, while the core@shell system shows moderate activity at room temperature, which can be significantly enhanced by increasing the temperature. In this section, we evaluate the performance of these materials as magnetically activated catalysts. Following, the ability of both the core and core@shell systems to degrade organic compounds under alternating magnetic field exposure (60 mT, 96 kHz) was assessed.

Figure 6a and b show the temperature profiles of dispersions under magnetic hyperthermia conditions for core and core@shell CS1 systems, respectively. Once the temperature stabilized at approximately 53 °C, hydrogen peroxide was added to start methylene blue degradation. The reaction medium consisted of nanoparticles dispersed in acetate buffer (pH = 5) at a concentration of 1 mg of NPs/mL, with an initial dye concentration of 100 ppm. The degradation kinetics of methylene blue under MH is presented in Figure 6c. Consistent with the results obtained using conventional thermal heating, the core Zn_0.2_Fe_2.8_O_4_ nanoparticles did not show activity. In contrast, the core@shell system achieves 99% dye degradation after 1 h of reaction.

**Figure 6:**
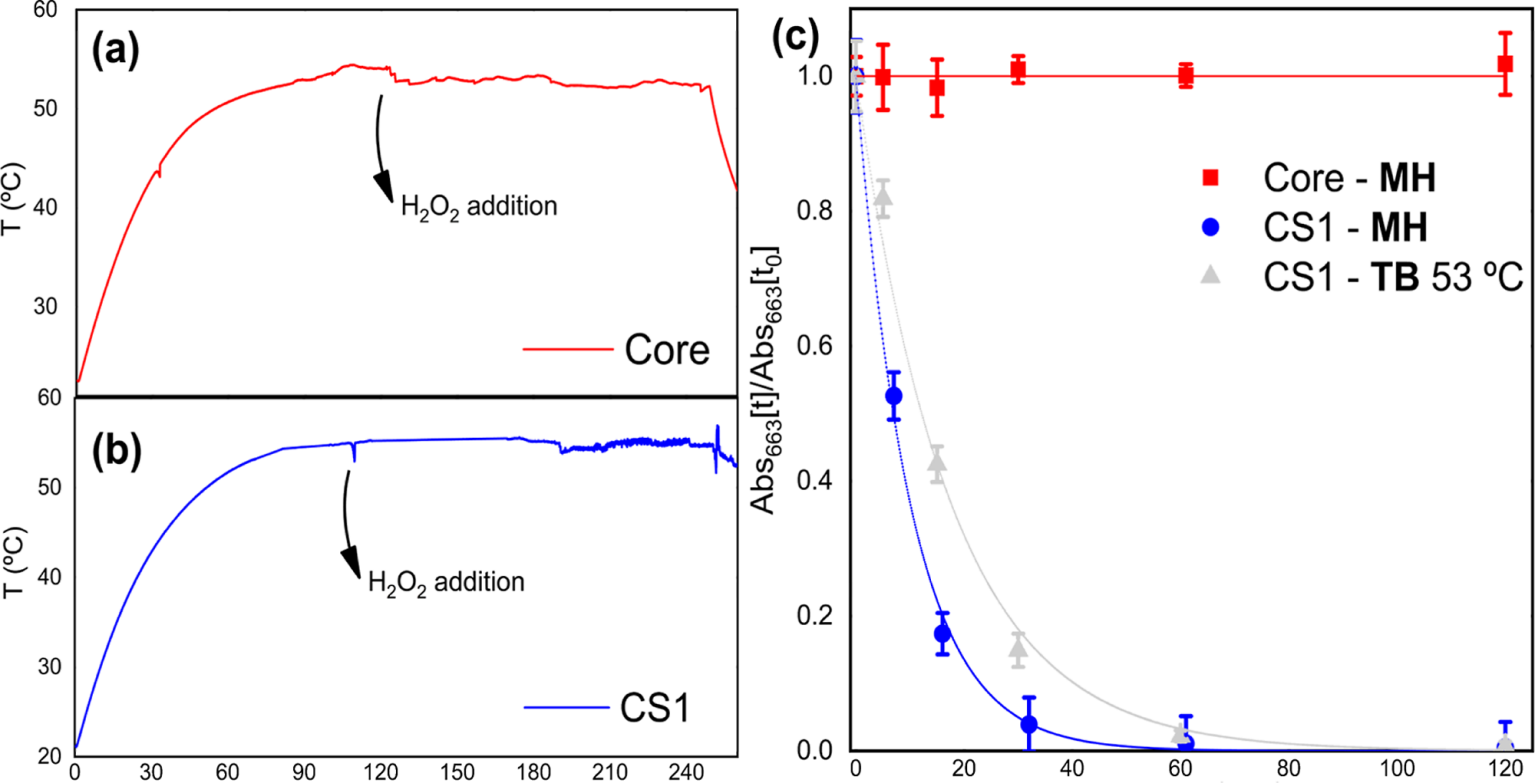
Magnetic-hyperthermia-driven degradation of 100 ppm of methylene blue, using 1 mg/mL of nanoparticles, 10 μL/mL of H_2_O_2_ in acetate buffer at pH 5, under an alternating magnetic field (60 mT and 96 kHz). Temperature–time profiles generated by (a) zinc ferrite nanoparticles and (b) CS1 core@shell system. (c) Normalized absorbance at 663 nm as a function of time (*t*) under MH conditions for the core (red) and core@shell (blue) systems and for the core@shell system under a thermal bath (TB, 53 °C). Solid lines correspond to pseudo-first-order (PFO) fits (see Figure. S3).

The comparison between the heating methods highlights a higher catalytic efficiency under magnetic hyperthermia conditions. When the core@shell system is heated up to 53 °C using a thermal bath, approximately 40% degradation is achieved within 15 min, whereas this efficiency increases to over 80% when the experiment is performed at the same temperature but heated by magnetic hyperthermia. To quantitatively compare both conditions, the degradation curves were fitted using a pseudo-first-order (PFO) kinetic model (Figure S3). The core@shell catalyst shows a higher kinetic constant under magnetic hyperthermia (*k*_MH_ = 0.0990(4) min^–1^) compared to conventional thermal heating at the same bulk temperature (*k*_TB_ = 0.0570(4) min^–1^). This enhancement in the rate constant can plausibly be attributed to the localized heating associated with magnetic hyperthermia heating.^48^ In this mechanism, each nanoparticle core acts as a nanoscale heat source, potentially promoting surface reactions more effectively than uniform bulk heating. As a result, the catalytic efficiency may be higher under magnetic hyperthermia even when the measured bulk temperature is comparable to that reached under conventional heating.

XPS measurements of the core and CS1 samples before and after the colorimetric experiments under magnetic hyperthermia conditions were performed to evaluate the catalyst stability. Figure S4 shows the high-resolution spectra of core and CS1 samples in the 50–100 eV binding energy region. The post-catalysis samples exhibit a significant decrease in the Cu 3p peak, suggesting the occurrence of leaching (Table 1). These results are supported by ICP measurements, were the Cu/Fe ratio decrease from 0.15 to 0.12 after one cycle, as presented in Table 1.Previous studies have reported that copper phases are readily attacked by H_2_O_2_ and easily undergo leaching.^49–51^ In addition, the incomplete transformation of Cu­(OH)_2_ to CuO could also facilitate the observed copper loss.

To evaluate the possible homogeneous contribution arising from metal leaching, additional colorimetric experiments were performed using the supernatant obtained after the magnetic separation of the nanoparticles. In these experiments, methylene blue and hydrogen peroxide were added to the recovered supernatant under the same temperature conditions, and the results are presented in Figure S5. The supernatant exhibits measurable catalytic activity, attributed to the presence of dissolved Cu and Fe ions. However, this activity is significantly lower than that of the core@shell catalyst. While the supernatant reaches about 38% discoloration after 1 h, the core@shell system achieves nearly complete degradation within the same period. Kinetic analysis shows that the apparent PFO rate constant of the core@shell system is approximately seven times higher than that of the supernatant (0.0570(4) min^–1^ vs 0.0080(1) min^–1^ for CS1 and supernatant at 53 °C thermal bath, respectively). These results indicate that although homogeneous reactions due to leached metal ions contribute to the overall process, they cannot account for the catalytic performance observed for the nanoparticle system.

These results demonstrate that integrating magnetic and catalytic materials within a core–shell architecture introduces additional functionalities to the system. The magnetic properties of the core not only enable magnetic recovery of the catalysts for subsequent reuse but also allow the generation of magnetic hyperthermia, which enhances the kinetics of the catalytic reaction and provides a means for remote enhancement of the process. However, the material presented here still requires further optimization to improve catalyst stability by minimizing metal leaching, thereby enhancing the reuse efficiency. In addition, improvements in the scalability of the synthesis route are also needed to meet the material demand and to reduce production costs.

## 4 Conclusions

In this work, we developed a Zn_0.2_Fe_2.8_O_4_@Cu­(II)-based core@shell nanocatalyst that exhibits magnetically enhanced Fenton-like catalytic activity through magnetic hyperthermia. The zinc ferrite core was engineered to efficiently generate heat under an alternating magnetic field, while the copper phase shell (CuO/Cu­(OH)_2_) provided Fenton-like catalytic functionality that increased activity at elevated temperatures.

EPR spectroscopy using the spin trapping technique confirmed that ·OH radicals were the primary reactive species generated during the reaction. While zinc doping in the ferrite core suppressed the radical production, coating with a CuO/Cu­(OH)_2_ shell significantly enhanced ·OH generation, consistent with the observed improvement in dye degradation.

Colorimetric assays using methylene blue revealed that the catalytic activity is highly dependent on the copper content. While the bare zinc ferrite NPs exhibited no catalytic activity, even at high temperatures, the core@shell systems achieved nearly complete degradation of methylene blue at 60 °C.

Under magnetic hyperthermia conditions, the core@shell nanocatalyst degraded 99% of the organic dye within one hour, whereas the bare zinc ferrite NPs remained inactive. Notably, a synergistic effect between catalytic activity and magnetic hyperthermia was observed; at comparable bulk temperatures, dye degradation was more efficient under magnetic hyperthermia than under conventional thermal methods. This behavior is likely related to the heating mechanism, where each nanoparticle acts as a localized heat source under an alternating magnetic field, potentially accelerating surface reactions.

Overall, these results highlight the potential of core@shell architectures for the design of multifunctional materials for magnetic hyperthermia-enhanced catalysis. This approach enables the independent optimization of the magnetic core for efficient heat generation and the catalytic shell, which shows negligible activity under ambient conditions but can be remotely enhanced by local heating under an alternating magnetic field. In addition, the magnetic core facilitates the magnetic recovery and reuse of the particles. This strategy provides a versatile platform to tailor catalytic performance and improve efficiency in targeted applications such as biomedical therapies and environmental remediation.

## Supplementary Material



## Notes

HAADF–STEM imaging and EDS elemental mapping of CS2 nanoparticles; zeta potential of core and CS1 nanoparticles as a function of pH; magnetic parameters of core and core@shell systems derived from hysteresis loops; pseudo-first-order kinetic analysis of methylene blue degradation catalyzed by core and CS1 nanoparticles under magnetic hyperthermia and thermal bath conditions; high-resolution XPS spectra of as-prepared and postcatalysis core and CS1 nanoparticles; and evaluation of homogeneous contribution to methylene blue degradation under a 53 °C thermal bath after catalyst removal (PDF)
