# Supplementary material for: Engineered Zn0.2Fe2.8O4@Cu(II)-Based Core@Shell Nanoparticles for Magnetic Hyperthermia-Enhanced Catalysis
Source: ACS Appl Nano Mater. 2026 Apr 24;9(18):8358–69. doi: 10.1021/acsanm.6c01226 (PMC13162199; doi:10.1021/acsanm.6c01226)
Supplement: Supplementary file 1 [file an6c01226_si_001.pdf]

Engineered $Zn_{0.2}Fe_{2.8}O_4$@Cu(II)-Based Core@Shell Nanoparticles for Magnetic Hyperthermia-Enhanced Catalysis

Nahuel Nuñez[1,2,3,4], Carlos Díaz-Ufano[5], Alvaro Gallo-Cordova[5], Francisco Javier Palomares[5], María del Puerto Morales[5]*, Elin L. Winkler[1,2,3]**

[1] Instituto de Nanociencia y Nanotecnología (CNEA-CONICET), Av. Bustillo 9500, (8400) S. C. de Bariloche (RN), Argentina.
[2] Departamento Magnetismo y Materiales Magnéticos, Gerencia de Física, Centro Atómico Bariloche, Av. Bustillo 9500, (8400) S. C. de Bariloche (RN), Argentina.
3 Institu to Balseiro, CNEA-UNCuyo), Av. Bustillo 9500, (8400) S. C. de Bariloche (RN), Argentina.
[4] Escuela de Doctorado UAM, Centro de Estudios de Posgrado, Universidad Autónoma de Madrid. C/ Francisco Tomás y Valiente, 2, 28049, Madrid, Spain.
[5] Instituto de Ciencia de Materiales de Madrid, ICMM/CSIC, C/ Sor Juana Inés de la Cruz 3, 28049, Madrid, Spain.
* nahuel.nuez@ib.edu.ar (N. N.), elin.winkler@ib.edu.ar (E. L. W.), puerto@icmm.csic.es (M. P. M.)

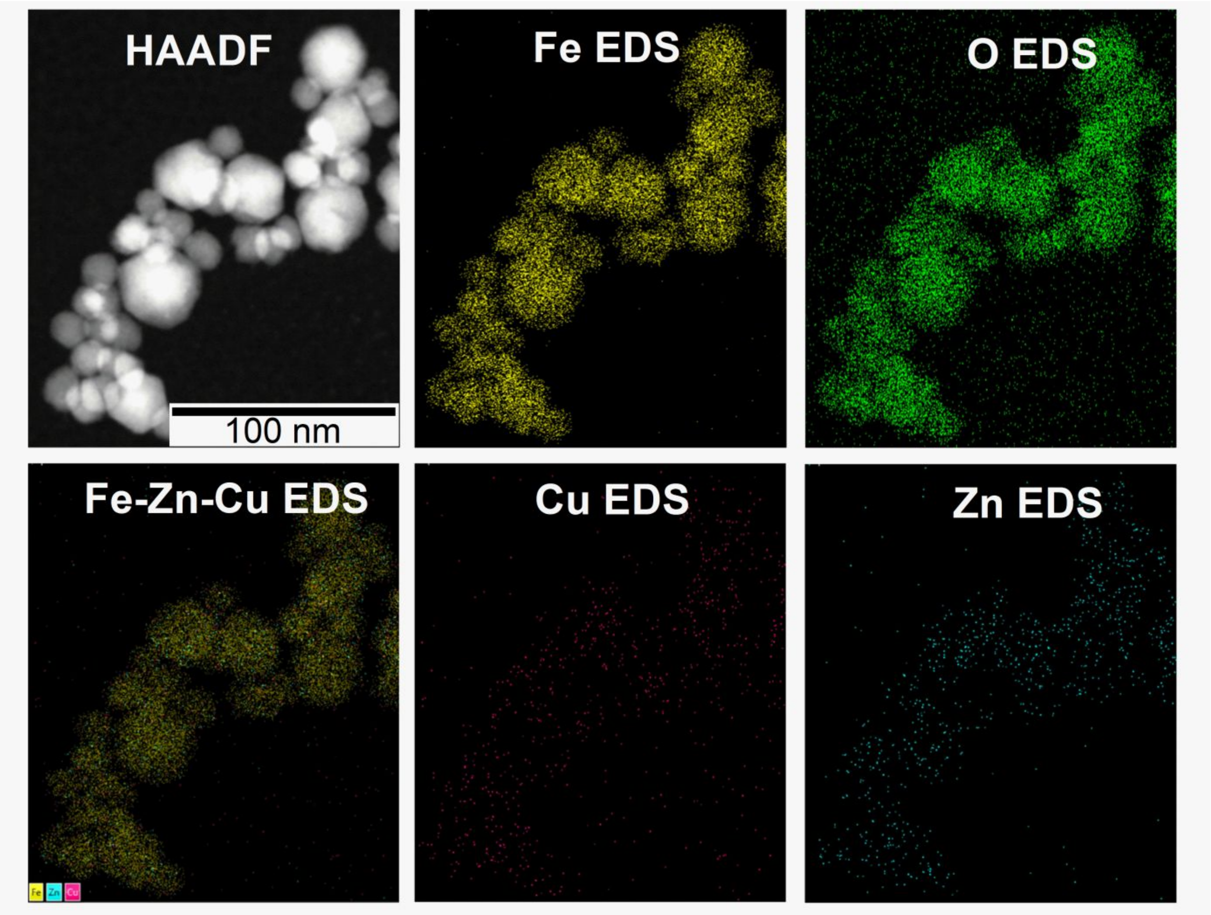

Figure S1. (a) HAADF image of the CS2 NPs. STEM compositional mapping of: (f) Fe, (g) O, (h) Cu and (i) Zn. The combined STEM compositional mapping of Fe, Cu and Zn is presented shown in (j).

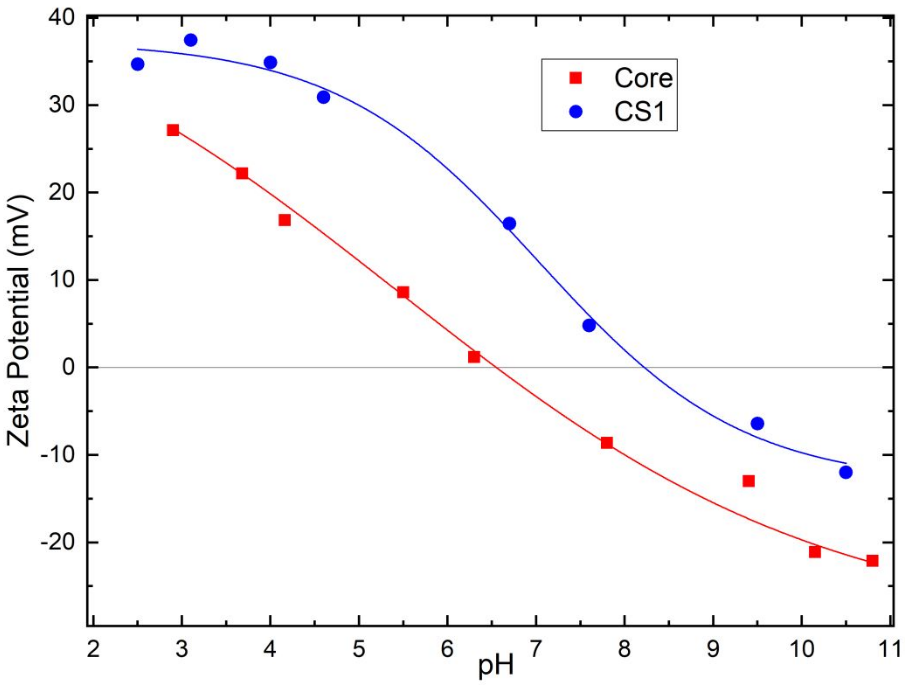

Figure S2. Zeta potential of core (red) nanoparticles and CS1 (blue) nanoparticles as a function of pH. The bare $Zn_{0.2}Fe_{2.8}O_4$ nanoparticles exhibit an isoelectric point near pH ≈ 6.5, whereas the core@shell system shows a shift toward higher pH values (≈ 8.5), consistent with the presence of a $CuO/Cu(OH)_2$ surface coating.

Table S1. Magnetic parameters of the core and core@shell nanoparticles obtained from room-temperature hysteresis loops presented in **Fig. 3a**. The table includes the shell to core mass ratio (m_{shell}/m_{core}) estimated by ICP, coercive field (H_c), saturation magnetization (M_s), remanent magnetization (M_r), and the M_r/M_s ratio. Values in parentheses indicate the uncertainty in the last digit.

	**Core**	**CS1**	**CS2**
**m_{shell}/m_{core}**	0	0.15	0.09
**H_c (Oe)**	26(1)	24(1)	19(1)
**M_s (emu/g)**	76(4)	61(3)	63(4)
**M_r (emu/g)**	6	4	4
**M_r/M_s**	0.08	0.06	0.06

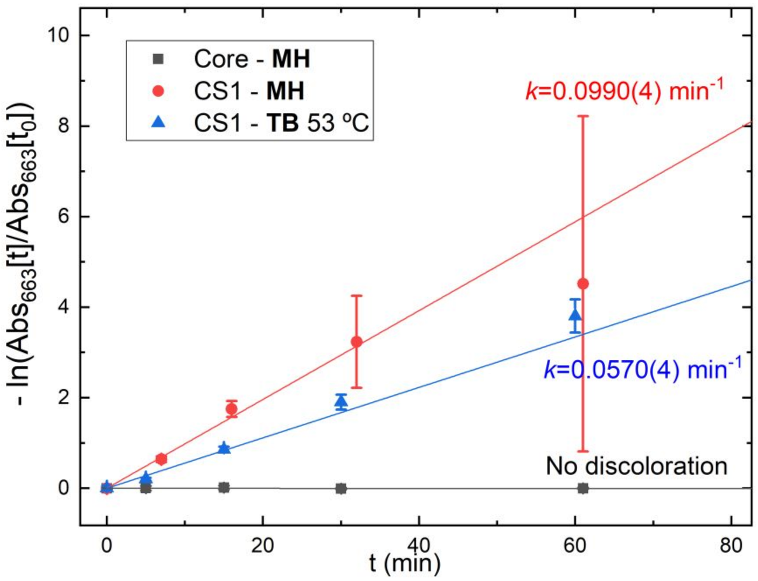

Figure S3. Pseudo-first-order kinetic analysis of methylene blue degradation catalyzed by CS1 nanoparticles under magnetic hyperthermia (MH, red) and thermal bath (TB, blue) conditions at a bulk temperature of ~53 °C. Linear plots of -ln(Abs_{663}[t]/ Abs_{663}[t_0]) versus time (t) are shown. The apparent rate constants obtained from the linear fits are k_{MH} = 0.0990(4) min^{-1} and k_{TB} = 0.0570(4) min^{-1} for CS1 system heated by MH and TB respectively. Zn-ferrite core nanoparticles does not show MB degradation.

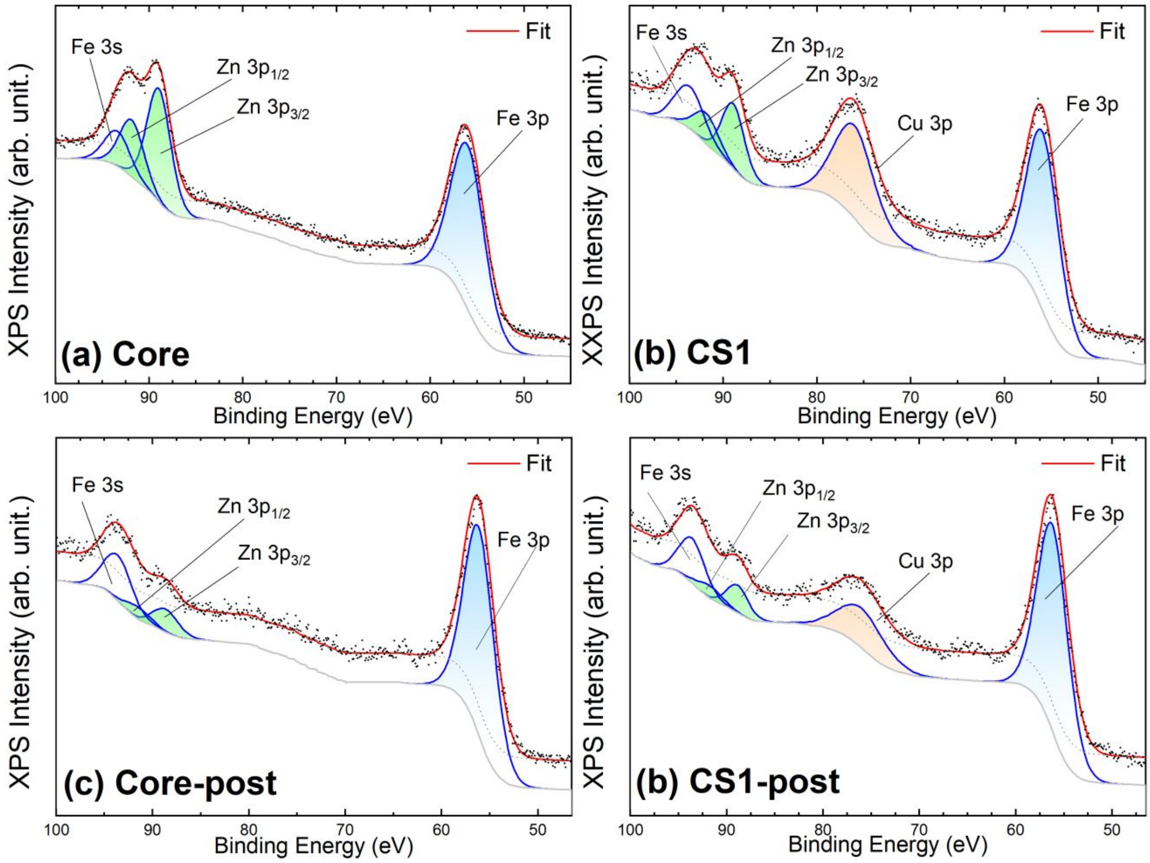

Figure S4. High-resolution XPS spectra of as-made (a) and used (c) $Zn_{0.2}Fe_{2.8}O_4$ (core) nanoparticles, and as-made (b) and used (d) $Zn_{0.2}Fe_{2.8}O_4$@Cu(II)-based core@shell (CS1) nanoparticles in the 50–100 eV binding energy region, highlighting the Fe, Zn, and Cu 3p core-level peaks.

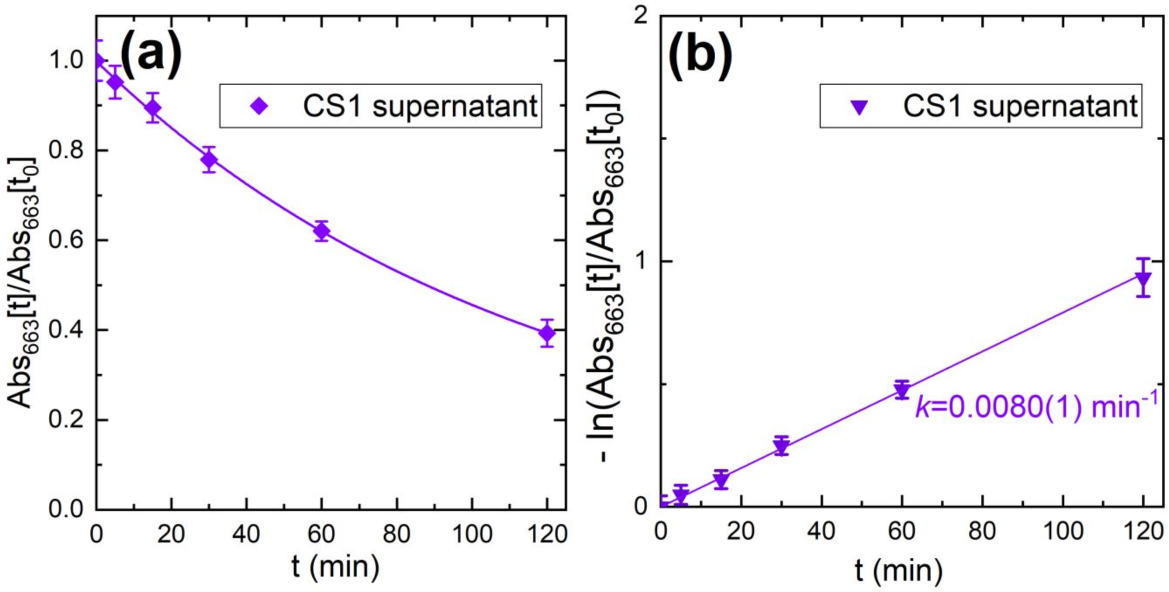

Figure S5. Evaluation of the homogeneous contribution to methylene blue degradation under 53 ºC thermal bath after catalyst removal. **(a)** Degradation kinetics of methylene blue in the supernatant obtained after magnetic separation of the $Zn_{0.2}Fe_{2.8}O_4$@Cu(II)based core@shell (CS1) nanoparticles. **(b)** Pseudo-first-order (PFO) kinetic analysis shown as linear plots of -ln(Abs_{663}[t]/Abs_{663}[t_0]) versus time (t). Reaction was performed at 100 ppm MB concentration, and 10 µL H_2O_2/mL.
